# Diversity Competency and Access to Healthcare in Hospitals in Croatia, Germany, Poland, and Slovenia

**DOI:** 10.3390/ijerph182211847

**Published:** 2021-11-12

**Authors:** Robert Doričić, Marcin Orzechowski, Marianne Nowak, Ivana Tutić Grokša, Katarzyna Bielińska, Anna Chowaniec, Mojca Ramšak, Paweł Łuków, Amir Muzur, Zvonka Zupanič-Slavec, Florian Steger

**Affiliations:** 1 Department of Social Sciences and Medical Humanities, Faculty of Medicine, University of Rijeka, 51000 Rijeka, Croatia; ivanatg@uniri.hr (I.T.G.); amir.muzur@uniri.hr (A.M.); 2 Institute of the History, Philosophy and Ethics of Medicine, Ulm University, 89073 Ulm, Germany; marcin.orzechowski@uni-ulm.de (M.O.); marianne.nowak@uni-ulm.de (M.N.); florian.steger@uni-ulm.de (F.S.); 3 Center for Bioethics and Biolaw, Faculty of Philosophy, University of Warsaw, 00-927 Warszawa, Poland; katarzyna.bielinska@uw.edu.pl (K.B.); anna.chowaniec90@gmail.com (A.C.); p.w.lukow@uw.edu.pl (P.Ł.); 4 Institute for History of Medicine, Faculty of Medicine, University of Ljubljana, 1000 Ljubljana, Slovenia; mojca.ramsak@guest.arnes.si (M.R.); zvonka.slavec@gmail.com (Z.Z.-S.); 5 Department of Public Health, Faculty of Health Studies, University of Rijeka, 51000 Rijeka, Croatia

**Keywords:** access to healthcare, healthcare inequality, health services, ethics, diversity, ethnicity, religious belief, sexual orientation

## Abstract

Diversity competency is an approach for improving access to healthcare for members of minority groups. It includes a commitment to institutional policies and practices aimed at the improvement of the relationship between patients and healthcare professionals. The aim of this research is to investigate whether and how such a commitment is included in internal documents of hospitals in Croatia, Germany, Poland, and Slovenia. Using the methods of documentary research and thematic analysis we examined internal documents received from hospitals in these countries. In all four countries, the documents concentrate on general statements prohibiting discrimination with regard to healthcare provision. Specific regulations concerning ethnicity and culture focus on the issue of language barriers. With regard to religious practices, the documents from Croatia, Poland, and Slovenia focus on dominant religious groups. Observance of other religious practices and customs is rarely addressed. Healthcare needs of patients with non-heteronormative sexual orientation, intersexual, and transgender patients are explicitly addressed in only a few internal documents. Diversity competency policies are not comprehensively implemented in hospital internal regulations in hospitals under investigation. There is a need for the development and implementation of comprehensive policies in hospitals aiming at the specific needs of minority groups.

## 1. Introduction

In recent years, the societal composition in many countries has been changing. Development towards more diverse societies has been observed as a result of migration, globalization, or changes in the age structure of the population. Additionally, increased sensibility concerning the existence of various social groups promotes a better understanding of the particular needs of these groups as well as shortages in the provision of appropriate services to them. Equal access to healthcare is one of the most important aspects of a diverse society and one of the main concerns for medical ethics. Disparities in access to healthcare have been observed in the case of various minority groups: migrants [1], patients from various cultural or religious groups [2], or with regard to gender, gender identity, or sexual orientation [3]. As a way towards addressing inequality in healthcare in Europe, the fundamental right to health and to access to healthcare has been legally enshrined in international treaties, including several European Union’s (EU) instruments, e.g., the EU Treaties or the European Charter of Fundamental Rights [4].

To improve access to healthcare, the concept of cultural competence has been proposed as a means to the provision of equal and high-quality healthcare and reduction of disparities among various groups of patients [5]. However, although this concept has been used in a number of research projects for more than two decades, there is still ambiguity regarding its definition or components. Most broadly, the concept can be characterized as the ability to work and communicate effectively and appropriately with individuals from different backgrounds [5,6]. More narrowly, and in reference to healthcare, it may be defined as a commitment to, or institutionalization of appropriate policies and practices to improve the capacity of healthcare systems, organizations, and healthcare professionals in the provision of quality healthcare for diverse populations [7,8]. However, the term ‘cultural competence’ itself can also imply a narrow understanding of the issue, as a concept that only applies to racial and ethnic minorities. Therefore, a more appropriate term ‘diversity competence’ has been proposed [9] (p. 170). It implies a broader perspective and inclusion of a wider population of minority groups, such as, but not exclusively, religious minorities, individuals with non-heteronormative sexual orientation, or individuals with different gender identities. We rely on this latter concept in our research.

Increasing societal diversity poses challenges for policy-makers, healthcare practitioners, and scholars for developing competent healthcare services with the potential to reduce healthcare inequalities. Diversity competence allows the provision of tailored healthcare adapted to the requirements of individual patients [10,11] increases the capacity of healthcare services to provide equitable and ethical care [12,13] and improves healthcare professionals’ insight and empathy in patients’ beliefs, values, experiences, and behavior [14,15]. Improved diversity competence can result in better-perceived quality healthcare, improved adherence to treatment, higher patient satisfaction, more effective interaction, and improved health outcomes [16].

Two elements of diversity competence in healthcare providers, e.g., hospitals, have been highlighted: practices and policies [17,18,19,20,21]. Practices involve individual interactions between healthcare professionals, e.g., doctors, nurses, medical assistants, and patients from diverse populations. Central components of diversity competent practice are knowledge, skills, and attitudes. However, these capabilities do not evolve on their own but are shaped in a healthcare institution context through organizational factors, such as policies, procedures, and allocation of resources [22]. They express general values, on which the healthcare organizations base their activities, guidelines for interactions in a specific situation, as well as intended actions to reach the goal of culturally responsive healthcare services, such as staff training, or provision of interpreters. Thus, they are of crucial importance as they provide the structural framework for diversity competent practice, which otherwise would only occur arbitrarily depending on the personal attitude of the healthcare professional. Diversity competence in healthcare organizations results in improved patient quality of life, patient satisfaction, and adherence to treatment requirements i.e., patient compliance as well as overall improvement in healthcare organization’s performance [17,18].

Up to now, analysis of diversity competence concentrated mostly on healthcare organizations in the USA, Australia, and Canada; similar examples from Europe, especially from Central and Southern Europe are rare [23,24]. They mostly consider diversity competence in the context of ethnicity or race, neglecting other characteristics of diverse societies. Moreover, according to our knowledge, there are no comparative studies, which focus on the approach towards diversity competence in the comparative perspective of healthcare organizations from various countries of Central and Southern Europe.

To fill out this research gap, the aim of this paper is to analyze policies and procedures regarding diversity competency in hospitals in four European countries: Croatia, Germany, Poland, and Slovenia. Comparison of these countries may be valuable for several reasons. First, they all belong to the EU and as Member States of this organization, they are subject to its binding normative instruments. However, in the context of national regulations, policies, or attitudes towards minority groups, healthcare organizations can display dissimilar sensitization towards the topic. Second, these countries display various sociocultural demographics, which also underwent significant changes in recent years, e.g., migration trends contributed towards more ethnically and religiously diverse society in Germany, but also in Croatia and Slovenia, which are migration-transition countries. Additionally, in Poland, the number of third-country nationals on permanent or temporary stay has risen significantly. Third, these countries demonstrate various levels of economic development, which could influence the allocation of resources towards diversity programs, training, or appointment of staff designated to administer various issues concerning minority groups. The key research question is: how has the issue of social diversity been addressed in hospitals’ internal regulations in these four countries? Through analysis of these regulations we evaluate organizational factors, that is policies and procedures, implemented in order to tackle the question of equality in healthcare for minority groups. In our attempt to answer this question, we have focused on the three following dimensions of diversity: (i) race and ethnicity, (ii) religion and belief, (iii) gender, gender identity, and sexual orientation.

## 2. Materials and Methods

**Figure 1:**
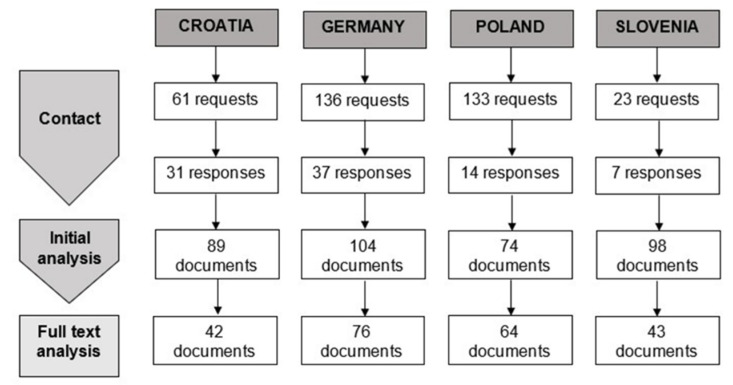
Procedure of the research.

The research was conducted by a multi-disciplinary and multi-lingual team of authors, representing various fields of expertise: bioethics and medical ethics, philosophy, public health, political science, history of medicine, anthropology, social work. In order to answer the research questions, we have conducted documentary research combined with thematic analysis of materials provided by healthcare institutions in Croatia, Germany, Poland, and Slovenia. The particular steps of the research are shown in Figure 1.

### 2.1. Procedure

Between June and November 2020, we have contacted hospitals in all four countries with a request for relevant internal documents. In the case of Germany and Poland, the hospitals to which the request was addressed were selected according to their size (small-, medium-, and large hospitals) and geographical location—targeted were hospitals in all regions of these two countries. Due to the smaller number of hospitals in Croatia and Slovenia, the request was sent to all public hospitals in these countries. The request was sent to different types of hospitals: university clinics, general and specialist hospitals, e.g., gynecological, psychiatric, pulmonary, orthopedic, and rehabilitation hospitals. Addressed were public and private hospitals with non-confessional and confessional providers. We did not send an invitation to private self-paying hospitals and those with a partial concession in the Slovenian case (Table 1).

Initial contact occurred through mail or electronic mail. In all four countries, we have sent an analogous letter in the native language to directors of the hospitals with the request for providing us with respective internal documents or for forwarding our request to the appropriate office. In case there was no response to our initial request, after several weeks we have repeated our attempt for contact through mail, electronic mail, or phone. In the case of phone contact, we have subsequently provided our request through electronic mail.

### 2.2. Materials

We rely on documents that were sent to us directly by mail or by electronic mail or in some cases on materials from hospital web pages specifically referred to us by the hospitals that were contacted. We have received 365 documents altogether. The materials encompass different categories of documents: statements, position papers, minutes of meetings, codes of ethics, policy documents, statutes, information or brochures for patients, charters of patients’ rights, organizational charts and manuals, information on hospitals’ websites, best practices examples, and lists of educational trainings on cultural diversity. Though very different in categories, all these documents meet the criteria required for sources of documentary research: authenticity, credibility, representativeness, and meaning [25] (p. 183).

### 2.3. Analysis

In the first step, we have conducted an initial evaluation of the materials. We have examined the texts of all documents to assess the relevance of the materials provided to the research questions. Excluded were 140 documents, which did not address the issue of diversity in healthcare or were not pertinent to the specific categories of diversity included in the research.

In the second step, thematic analysis was performed. Thematic analysis is a qualitative descriptive approach, for identification, analysis, and reporting of common patterns or themes that extend throughout a set of the analyzed material [26]. Four thematic categories pertaining to the research question were deductively formed: (1) general anti-discrimination statements; (2) ethnicity, race, and culture; (3) religion and belief; (4) gender, gender identity, and sexual orientation. These categories are intended to present the most important aspects contained in the documents. It is important to note that the categories are not mutually exclusive—the content of documents presented in some of the categories may overlap with other categories.

The thematic analysis of the full text of documents pertinent to the research question was conducted. Specific statements from the documents were coded manually in order to identify the data that matched the pre-defined categories, determine possible sub-categories, or expand the catalog of these categories for new items. The coding process involved highlighting relevant information with notes on the text. This information was then extracted, assigned to particular categories, and grouped in thematic tables comprising all four countries. Quotes to illustrate the information were translated from Croatian, German, Polish, and Slovenian into English and placed in the tables. The data gathered in all categories were analyzed to differentiate common patterns, topics, as well as similarities and differences between thematic content in particular countries [27]. Drawing on the key findings from the analysis, we developed a narrative synthesis of the results.

## 3. Results

We have received altogether 94 responses to our inquiry. In Croatia, the response rate was 50.8%, in Germany 27.2%, in Poland 10.5%, and in Slovenia 30.4%. Altogether, we received 365 documents.

The topic of equal access to healthcare and anti-discrimination in healthcare is variously covered in the internal documents received from hospitals in all four countries. In the following, we present the identified content in the four categories.

### 3.1. General Anti-Discrimination Statements

Table 2 shows different groups of anti-discriminatory statements found in the hospitals’ internal regulatory documents. The hospital documents either explicitly prohibit discrimination or the anti-discrimination provisions are mentioned indirectly, through various principles on equality. The category of anti-discriminatory statements is the most commonly addressed topic among received documents. In documents received from German (N = 20) healthcare institutions, anti-discrimination is stated as a guiding principle for activities and relations with patients. Additionally, non-discrimination as one of the guiding principles can be found in institutional codes of ethics (N = 4) received from Polish hospitals. Several of documents from German hospitals (N = 6) specifically refer to the German General Equal Treatment Act (Allgemeines Gleichbehandlungsgesetz), which aims to eliminate discrimination among others in the specific area of healthcare or in relations between employer and employee. Similarly, the hospitals’ internal documents from Croatian (N = 2), Polish (N = 5) and Slovenian (N = 1) health institutions contain anti-discrimination provisions for employees. Anti-discrimination provisions concerning healthcare users are presented directly or indirectly. Direct provisions are contained in codes of ethics obtained from Polish hospitals (N = 4). Numerous documents from Polish hospitals included indirect provisions, reflecting legal guarantees of equal access to healthcare services funded from public funds and patient’s rights (Patient’s Rights Act as a legal base for the organizational regulations N = 8, direct quotation of the Act’s contents N = 3). Same indirect provisions were included in the internal documents from Croatian hospitals (N = 6). In Slovenia, general and specific anti-discrimination provisions are part of the health and anti-discrimination laws, which are indirectly mentioned in hospital documents (N = 2) related to patients’ rights.

### 3.2. Ethnicity, Race, and Culture

As can be seen in Table 3, obtained results on the statements regarding ethnicity, race, and culture can be divided into three major topics: (1) language barriers; (2) improvement of access for ethnic or cultural minority groups, and (3) integration of foreign workers into the hospital’s staff. The central issue within this category is the subject of language barriers between patients and healthcare professionals (Table 3). It has been recognized in several documents in all four countries. Health institutions in our sample regulate that issue through various procedures. In Croatia, healthcare institutions (N = 2) provided a list of official interpreters, in case of patients’ lack of knowledge of the Croatian language. In Germany, the documents (N = 5) indicate language barriers as obstacles in the treatment of patients with limited or no command of the German language. Responding healthcare institutions (N = 4) provided information about the establishment of interpreting services. One hospital mentions the involvement of the entire healthcare professional team in cooperation with the translator. A similar approach can be identified in documents received from hospitals in Poland (N = 8) and Slovenia (N = 1), which identify the employees who speak a foreign language as the main form of provision of interpreting support. Additionally, mentioned is the possibility of involvement of interpreters from other institutions, for example, embassies, consulates, or an interpreting company.

Apart from language barriers in the healthcare context, German hospitals (N = 2) included examples of institutional commitment towards the improvement of access for ethnic or cultural minority groups. This occurs through a provision of specialized service by a trained person, a so-called ‘guide’. One German hospital provided information on training for employees on interactions with Muslim patients. The document from one Slovenian hospital shows that this institution organizes once a year a training for employees on patients’ rights.

German hospitals (N = 4) provided information on the integration of foreign workforce into the hospital’s staff. Received documents point out the necessity of support for foreign employees through a dedicated integration manager or in the form of language courses, dispute resolution, or intercultural mediation instruments. No such statements about inclusion of foreign workers into staff could be found in the documents received from Croatian, Polish, and Slovenian hospitals.

### 3.3. Religion and Belief

Results regarding religion and belief can be divided into three topics: (1) the pastoral care, (2) respect for customs and practices, and (3) meeting religiously motivated needs. Then, regarding pastoral care (1) three issues have been identified: (a) access to clergy, (b) space dedicated for religious practices, (c) religious services provided. Concerning respect for other customs and practices, a specific field identified was diet. With reference to meeting religiously motivated needs (3), attention should be paid to (a) blood transfusions and blood products in case of Jehovah’s Witnesses patients (b) religiously motivated male circumcision (Table 4).

In Germany, healthcare institutions with confessional carriers (N = 7) point out in their mission statements Christian values and principles that should be reflected in all daily activities. However, these documents do not specify particular norms or codes of conduct based on these principles. Documents from Slovenian hospitals (N = 3) mention ‘religious spiritual care’. It is defined in reference to the Rules on the Organization and Provision of Spiritual Care in Hospitals and Other Health Care Providers (Pravilnik o organizaciji in izvajanju verske duhovne oskrbe v bolnišnicah in pri drugih izvajalcih zdravstvenih storitev), such as for example visiting and providing spiritual accompaniment to patients or providing religious rites for deceased patients. One hospital in Slovenia explicitly states in its internal regulation that healthcare is considerate and respectful of the patient’s personal values and beliefs and that each patient brings their own values and beliefs into the healthcare process. This hospital also states that every healthcare professional should strive to understand the care and services they provide within the perspective of the patient’s values and beliefs. A similar statement was found in internal documents from one Croatian hospital.

With regard to the practice of religious rites in healthcare institutions, religious service in a hospital chapel for members of the Roman Catholic faith is available in some Croatian (N = 2), German (N = 2), Polish (N = 8), and Slovenian (N = 2) hospitals. In the documents from Polish healthcare institutions, the legislative guarantee of patient’s right to pastoral care, regardless of their religion or belief, is articulated in various manners. They specify a provision of pastoral care for members of the Roman Catholic faith on a regular basis (N = 9) and the availability of pastoral care for other religions, mainly on request (N = 9) and often with contact lists provided. Hospitals in Slovenia (N = 3) provide patients with a list of contact persons from minority religious communities for pastoral care.

Further analysis of the received documents regarding religious customs and practices shows concentration on two specific issues—refusal of blood transfusion in the case of Jehovah’s Witnesses and male circumcision. In Croatia, one health institution has provided an opinion issued by the Ministry of Health that, in case of life-threatening conditions, the health care professionals should act according to professional standards and are not obliged to respect Jehovah’s Witnesses’ refusal of blood transfusion. Another institution in Croatia acts in accordance with the opinion of the internal Ethics committee (N = 1). Three of the responding healthcare institutions in Germany (N = 3) point out to respect of the patient’s will and explicit informed consent of the patient to blood transfusion in case of emergency. Another specific example of religious practice is male circumcision. One responding institution from Germany specifically includes this procedure in its guidelines, stating that circumcision is necessary for Jews and Muslims to preserve a religious identity and religious socialization of the child. However, employees of this institution have a right to refuse participation in it without any further justification. Documents from Slovenian hospitals (N = 2) show that male circumcision was available until 2012. One Slovenian hospital in its document reports that since 2012, ritual circumcision has been unacceptable in Slovenian hospitals for legal and ethical reasons, therefore in areas with a mixed population, including a Muslim population, the number of unprofessionally performed circumcisions increased until 2014, or they were performed in neighboring Austria. It can still be performed in one hospital as a self-paid service. In Croatia, the issue of male circumcision has not been addressed in the received internal documents. In Poland, no internal documents relating to these special needs have been identified.

Observance of other religious practices and customs is rarely addressed – the availability of the Halal diet is mentioned in only two documents (one from Croatia and one from Germany). Documents provided from German hospitals (N = 4) state that there is a possibility of religious accompaniment of the dying patient irrelevant of personal belief. It could be e.g., ritual ablutions for deceased Muslims in the case of German hospital. In Poland, it is guaranteed by the Patient’s Right Act and this guarantee is reflected by the hospital documents (N = 7).

### 3.4. Gender, Gender Identity, and Sexual Orientation

Several of the documents from Croatian (N = 1) and German (N = 10) hospitals point to gender-specific equal treatment; however, mostly concerning the employment environment and the issue of sexual harassment. According to these, sexual harassment represents the predominant issue regarding gender as a ground for discriminatory practices. One document from Germany states that sexual harassment constitutes a violation of human dignity and can cause serious health problems for the affected persons. Only one document, obtained from a Croatian hospital, explicitly mentions respect for specific needs in healthcare from a gender perspective.

In the documents obtained, the topic of gender identity is explicitly addressed in only one material received from a German hospital. In form of a short video, available in German and English, healthcare professionals are sensitized towards interactions with trans- and intersexual patients. 

German healthcare institutions (N = 5) explicitly mention sexual orientation in the context of healthcare. Two institutional statements about the concept of diversity and inclusion contain declarations ensuring equal treatment and respect for everyone regardless of sexual orientation. Other documents (N = 3) prohibit harassment or unequal treatment in the work environment based on sexual orientation.

General anti-discrimination provisions, regarding sexual orientation, but not gender identity are contained in institutional codes of ethics obtained from Polish hospitals (N = 5) and from hospitals in Slovenia (N = 1). Specific healthcare needs of LGBTQI+ persons are not mentioned in the received internal documents from Croatian, Polish, and Slovenian hospitals.

## 4. Discussion

Our findings show that the majority of hospitals’ internal documents contain only general statements prohibiting discrimination without specifically addressing needs of individuals with various ethnic or religious background or with regard to sexual orientation and gender identity. In the context of ethnicity, hospitals’ internal policies concentrate on language barriers. The documents analyzed differ considerably on issues of religious diversity. Documents from Croatia and Germany rarely mention the customs of religious minorities and the needs of pastoral care. In contrast, numerous documents from Poland and Slovenia deal with the pastoral care of patients with a non-dominant religious background. Specific instruments for facilitation of inter-cultural competency are rarely implemented.

As indicated at the beginning of this paper, interactions with diverse social groups in a hospital setting are not only shaped by interactions between healthcare professionals and healthcare users but also through policy frameworks and organizational arrangements of the healthcare organization [28]. Without organizational commitment, sustained change in attitudes of healthcare professionals is difficult to achieve. Literature on the topic highlights the role of internal policies as an intervention strategy for the improvement of cultural/diversity competency in healthcare organizations [29]. The aim of such intervention is to modify the practices of healthcare organizations. It can be accomplished through various measures, such as diversity protocols or policies, provision of interpreter services and translation of materials, workforce diversity, workforce training, or tailored programs for healthcare users.

Most common in the analyzed documents are policy statements concerning inclusion and anti-discrimination of patients with various characteristics. This indicates organizational readiness for the provision of high-quality healthcare for members of minority groups. Implementation of clear mission and goals declarations is one of the factors, which can substantially improve diversity management in healthcare institutions [30,31]. However, an improvement in diversity competency requires a focused and systematic approach, such as integrating diversity competency into strategic planning, the dedication of staff and resources, or specific recruitment practices of the healthcare personnel [17]. Our analysis shows that such an approach is missing in most of the responding hospitals.

In several cases, visible in the provided material is that hospitals in designing their internal documents base their content or explicitly mention national normative framework. Examples include the General Equal Treatment Act in Germany, Patient’s Rights Act in Poland, Rules on the Organization and Provision of Spiritual Care in Hospitals and Other Health Care Providers in Slovenia. This points out the importance of national policies in addressing the questions of equity and antidiscrimination in healthcare. Such policies can provide a structure and support in application of diversity competence. It is however important that local healthcare institutions retain flexibility in implementation of the top-down policies, allowing them consideration for local context, situation, and resources. 

In dealing with issues of ethnicity and culture, the main emphasis in the analyzed documents is put on the language barriers, which hinder successful communication in the healthcare professional-patient setting. The provision of interpreter services has been identified in the literature as a factor considerably contributing to a better quality of care and positive health outcomes [32]. The responding hospitals mostly use ‘ad hoc’ interpreters, external interpreting services, or translations by health staff if they know the language, which can be used if necessary. The use of multilingual hospital employees as an interpreting resource is widespread due to availability and low cost; however, it cannot be a substitute for on-site, well-trained medical interpreters [33].

Only two responding hospitals, both from Germany, implemented tailored services for healthcare users in the form of trained persons—guides or advisors for migrants. The lack of knowledge of the entitlements or available services can be a major obstacle in the assessment of healthcare and appropriate use of services [34]. Difficulties in navigating the healthcare system due to poor understanding of administrative procedures or the appointment system are further impediments for patients, especially for migrants and refugees [35]. The organization of a dedicated service for intercultural mediation, as well as administrative support, can provide a double advantage. On the one hand, it can supply patients with information on their healthcare rights and healthcare availability. On the other hand, it can support healthcare providers in contacts occurring in a multicultural environment but also clarify the legal status and entitlements of individual patients; thus, decreasing the risk that these patients will be overlooked in the healthcare system.

Cultural or diversity competency training and workforce diversity are two strategies aiming at improving the diversity competence of health services and healthcare professionals. They are repeatedly mentioned in the literature on the topic [29,36]. Competence training can encompass various approaches: from teaching broadly applicable knowledge and skills for communication in the multi-cultural context, through training about certain characteristics, beliefs, and behaviors of relevant populations, to foreign language training. Such training can be provided through various methods, e.g., with the help of cultural experts or through learning directly from patients about their sociocultural perspectives and encounters with healthcare professionals [37]. Employing staff representing diverse backgrounds can contribute to the reduction of minority patients’ anxiety and enhance communication. Even in situations, in which such members of staff only accompany patients in their encounter with a physician, their presence can ensure patients’ comfort and confidence [31]. 

With regard to the issue of gender, gender identity, and sexual orientation, visible in the obtained samples is concentration on the subject of harassment in the work environment. Specific healthcare needs of patients with non-heteronormative sexual orientation, intersexual, and transgender patients are explicitly addressed in only a few internal documents. Yet, LGBTQI+ individuals can experience both overt and covert discrimination in healthcare, which leads to a disproportionate impact on their physical and mental health [38]. Specific competencies for practice with this population of patients require tailored responses to their health needs and discriminatory or exclusionary behaviors [39].

The findings of this research highlight the need for further research addressing diversity competence in healthcare organizations. First, our findings demonstrate the need to evaluate diversity competence in a wider perspective of various healthcare providers, e.g., private practices, primary and specialist healthcare institutions. Second, they call for a comprehensive assessment of diversity competence not only according to the normative regulations of healthcare organizations but also encompassing the realization of these norms in healthcare practice. Third, the findings can provide a basis for further research on the degree of diversity, competency, and patient satisfaction levels.

Our research has also several practical implications. First, it demonstrates the need for the development of specific institutional policies aiming at improving diversity competency. Research has shown that introduction of such policies results in increased access to health services, improved client and family satisfaction, and improved health outcomes, e.g., compliance with medication or uptake of prevention strategies such as health monitoring [17,18,29,36,40,41,42]. This aim should be addressed on both political and institutional levels. On the political level, state healthcare sectors need to take under consideration the changing composition of contemporary societies and, in accordance, develop specific policies, regulations, or guidelines towards the goal of equal access to healthcare for minority groups. This could take place through a requirement for establishment of specialized boards for consultation and training within healthcare institutions as well as allocation of specific resources for their functioning. Similar actions in the healthcare sector occurred in all four countries; for example, establishment of institutional Research Ethics Committees is an illustration of a successful creation of specialized boards in the area of clinical research. On the institutional level of healthcare institutions, attention should be paid to the specific requirements of particular patients’ groups in individual institutions. Important is the development of internal policies aiming at reporting specific difficulties and the introduction of specific actions towards alleviating these obstacles. However, from the ethical point of view, important is that the allocation of institutional resources toward this goal should not occur through decreasing resources intended for medical care. Rather, contributions of various stakeholders, i.e., health policy-makers, professional organizations, hospital organizations should be assigned.

Second, it shows the importance of the comprehensiveness of such policies. Most analyzed documents only generally address the issue of societal diversity, without focusing on the specific needs of particular minority groups.

Third, visible is the need for practical implementation of policies, through training programs, ethical or cultural counseling for healthcare professionals, or through organizational adaptation, that is provision of translators of administrative personnel responsible for assistance in accessing healthcare for vulnerable minority groups. Cultural competency training can lead to improvement in physician-patient communication [43,44,45]. Use of interpreters is connected with uptake in clinical and preventive health services [46]. Additionally, interventions in form of bi-lingual community workers were positively evaluated and led to an increase in screening rate, improved health behavior and status, or improved health knowledge [47,48,49]. However, a closer investigation of the translation of institutional policies into practical patient care requires further investigation [42].

## 5. Limitations

The findings of this research need to be considered in light of their limitations. First, the response rate to our inquiry shows various numbers for particular countries. The main reason for the varying response rate might be the period during which our research has been conducted. Our contact with hospitals occurred during the COVID-19 pandemic in all four countries under research. Healthcare professionals but also administrative personnel were under exceptional stress and time constraints. Although we have repeated our initial request in the case of the hospitals that did not respond, our request might not receive similar attention as it would in usual circumstances. As the trajectories of the pandemic were different depending on the country, response rate might mirror the possibility of hospitals to cooperate with our research. The second limitation is response bias. It can be assumed that received responses were provided by hospitals with established policies and procedures for diversity competency. Hospitals without such policies might have chosen not to respond to our request. The third limitation is that, as we based solely on what hospitals sent us, the sets of documents in the case of each institution varied, sometimes significantly. It is possible that some hospitals under examination have some other relevant documents. However, as neither were they sent to us nor were we referred to them in the response, they were not included in the analysis.

Because of the size of the sample, we cannot generalize our results to other hospitals in the countries under research. Hospital regulations and guidelines pertaining to the research question can exist in healthcare institutions that were not included in this research sample. Nevertheless, the size of the sample provides also an advantage—it allows detailed inspection of internal documents in hospitals that responded to our call. Moreover, we have analyzed only one aspect of diversity competency, namely policies and procedures in hospitals. Notwithstanding this, our research provides important points concerning the inclusion of particular topics or specific regulations in the internal documents of hospitals in the research countries. Because of the small sample size and structural or healthcare policy differences in all countries under analysis, the results of our investigation need to be triangulated in further research. This could be achieved through examination of diversity competence in healthcare practice in interviews with healthcare professionals or through surveys. Such examination can provide more precise results and will allow to develop more specific conclusions for healthcare policies and practices.

## 6. Conclusions

Disparities in access to healthcare are nowadays observed for various minority groups and are one of the main concerns for medical ethics and healthcare at all. Organizational factors, such as policies, procedures, and allocation of resources can increase diversity competency of healthcare organizations and improve care and patient satisfaction in healthcare. The results of our study show that such policies are not comprehensively implemented in hospital internal regulations in all four countries under investigation. The majority of documents contain general statements prohibiting discrimination without specifically addressing needs of individuals with various ethnic or religious backgrounds or with regard to sexual orientation and gender identity. Although several documents concentrate on language barriers in the patient-healthcare professional relationship, the identified approaches concentrate on the use of internal resources, rarely involving specialized interpreter services. Moreover, visible is the lack of diversity training programs and organization of dedicated service for intercultural mediation and administrative assistance for refugees. Furthermore, specific healthcare needs of patients with non-heteronormative sexual orientation, intersexual and transgender patients are not explicitly addressed. Therefore, it is necessary that healthcare organizations recognize the need for specific support instruments targeting various minority groups. Implementation of instruments such as diversity training programs and specialized interpreter or cultural mediator services can improve healthcare provision for disadvantaged social groups.

## Figures and Tables

**Table 1:** Categories with numbers of hospitals in each country to which the request was addressed.

	Croatia	Germany	Poland	Slovenia
University clinics	5	21	20	2
General and specialized hospitals	56	115	108	21
Confessional carrier	1	41	5	-
Non-confessional carrier	60	95	128	23

**Table 2:** Anti-discrimination statements in hospitals internal documents in Croatia, Germany, Poland, and Slovenia.

Anti-Discrimination Statements		
Anti-discrimination as a guiding principle	Anti-discrimination provisions for employees	Croatia, Germany, Poland, Slovenia
	Prohibition of discrimination on grounds of race, ethnic origin, nationality, religion or belief, gender and gender identity, sexual orientation, disability, age, political views, or social status	Germany, Poland
Indirect mention of anti-discrimination	Equal access to healthcare for all social groups Equal right of everyone to quality healthcare	Croatia, Poland Slovenia
	Equal treatment of all co-workers and patients Key principles	Poland, Slovenia

**Table 3:** Statements regarding ethnicity, race, and culture in hospitals internal documents in Croatia, Germany, Poland, and Slovenia.

Ethnicity, Race, and Culture		
Language barriers	‘Interpreter pool’ from among the employees of the institution or interpreting services	Germany, Poland, Slovenia
	Possibility of involvement of interpreters from other institutions	Poland, Slovenia
	Lists of official interpreters	Croatia
Improvement of access for ethnic or cultural minority groups	Support for migrant patients through administrative procedures	Germany
	Training for employees concerning medico-ethical challenges in interactions with patients	Germany, Slovenia
Integration of foreign workers into the hospital’s staff	Recognition of various backgrounds and experiences of foreign workers.	Germany

**Table 4:** Statements regarding religion in hospitals internal documents in Croatia, Germany, Poland, and Slovenia.

Religion and Belief				
Pastoral care	Access to clergy	On the regular basis	Pastoral care for the members of the Roman Catholic Church	Poland, Slovenia
		On request	Provision of the pastoral care for members of religious communities	Germany, Poland Slovenia
	Space dedicated for religious practices		Religious rites in a hospital chapel for members of the Roman Catholic faith	Croatia, Poland, Slovenia
			Possibility of ritual rites in a hospital chapel/dedicated space for members of other religious communities	Slovenia, Poland
	Religious services provided		Masses in hospital chapels, confession opportunities	Croatia, Germany, Poland, Slovenia
Respect for customs and practices		Diet	Halal diet	Croatia, Germany
		Other	A possibility of religious accompaniment of the dying patient	Germany, Poland
Meeting religiously motivated need		Jehovah’s Witnesses —blood transfusion	Acting according to professional standards and no obligation to respect refusal of blood transfusion	Croatia
			Respect of the patient’s will and explicit informed consent regarding refusal of blood transfusion	Germany
		Male circumcision	Necessity for religious identity and religious socialization of the child for Jews and Muslims	Germany
			Possibility of male circumcision as a self-pay service	Slovenia

## Data Availability

All data analyzed during this study are available on request from the corresponding author.
